# Hydrocephalus: A Review of Etiology-Driven Treatment Strategies

**DOI:** 10.7759/cureus.68516

**Published:** 2024-09-03

**Authors:** Sarah A Mirkhaef, Lauren Harbaugh, Gurjit Nagra

**Affiliations:** 1 Pathology, Arkansas College of Osteopathic Medicine, Fort Smith, USA; 2 Pathophysiology, Arkansas College of Osteopathic Medicine, Fort Smith, USA

**Keywords:** ventricular pressure, parenchymal pressure, choroid plexus, beta-integrins, intracranial pressure

## Abstract

Hydrocephalus is a broad term usually understood as cerebrospinal fluid (CSF) accumulation resulting in cerebral ventricular system expansion. The production of CSF is by the choroid plexus in lateral ventricles, flowing between the third and fourth ventricles and eventually to the subarachnoid space. It is critical for proper neuronal function. Hydrocephalus is a neurological pathology linked to high morbidity from neurocognitive and motor impairment. It is classified as either communicating or non-communicating. Communicating hydrocephalus is understood as a deficit at cranial arachnoid villi and granulation absorption sites. However, there has been evidence that extracranial lymphatic vessels in the ethmoid bone region also play a role, as indicated by decreased lymphatic absorption in rat models of hydrocephalus. Treatment typically involves surgical shunt placement or endoscopic third ventriculostomy (ETV) technique with or without choroid plexus cauterization (CPC). These surgical interventions have high failure risks and complications that require re-intervention, further increasing morbidity and mortality risks. To date, there are few nonsurgical treatment strategies, but many have proved limited benefit, and many patients still require surgery. This analysis lays out the typical treatments and explores new, innovative interventions by highlighting the active role of brain parenchymal tissue in the pathogenesis of hydrocephalus.

## Introduction and background

Hydrocephalus refers to excessive cerebrospinal fluid (CSF) accumulation within cerebral ventricles [1]. The condition is most frequently seen in infants, secondary to congenital malformations and intraventricular hemorrhage observed in premature babies [1]. Studies have estimated that around 40% of primary hydrocephalus has a genetic basis [2,3]. Additionally, pediatric hydrocephalus has an incidence rate of approximately 0.1-0.6% of live births, likely due to intraventricular hemorrhage in premature infants [3,4]. Nonetheless, posthemorrhagic hydrocephalus is not just in premature infants; it can also be seen in older populations due to the increased risk of stroke associated with comorbidities, such as hypertension or dyslipidemia, affecting the vasculature. 

However, the incidence rate of pediatric hydrocephalus is significantly higher in low-income countries because of an increased occurrence of neural tube defects and postinfectious hydrocephalus [5,6]. Hydrocephalus incidence is 123 per 100,000 births in low-income countries versus 79 per 100,000 births in high-income countries, making it a common congenital defect globally [6]. The mortality rate of untreated hydrocephalus is high at around 20%-87%, and treated hydrocephalus has a high cost of care due to work-up, treatment, equipment, follow-ups, and complication management [6]. With this high incidence rate, the largest disease burden in low-income countries, and the high cost of management compounded by death if left untreated, it is critical to continue understanding the pathophysiology and etiology behind hydrocephalus and use the knowledge to inform treatment. This paper discusses the traditional clinical treatment options for hydrocephalus and explores promising interventions informed by etiology. There are many complications associated with traditional treatment strategies; consequently, this review aims to understand the pathophysiology of hydrocephalus and draw connections between brain parenchymal tissue and hydrocephalus pathogenesis to explore future non-invasive interventions. 

Methods

Articles were gathered using PubMed and Google Scholar. PubMed and Google Scholar search terms included: hydrocephalus clinical treatment, non-surgical hydrocephalus treatment, memantine hydrocephalus, acetazolamide hydrocephalus, prevalence of pediatric hydrocephalus, TRPV4 antagonist hydrocephalus, SPAK inhibitor hydrocephalus. There were no exclusion criteria regarding the type of article, as reviews, books, meta-analyses, clinical trials, and original studies were reviewed from any publication year. The pieces were reviewed for relevance to hydrocephalus and therapeutic approaches. This review will highlight etiology followed by pathophysiology, including the role of aquaporins and their connection to clinical treatments. We will address problems with traditional surgical interventions. Lastly, we will highlight the non-surgical interventions used for hydrocephalus treatment, including acetazolamide, memantine, TRPV4 antagonist, NKCC1/SPAK inhibitors. 

## Review

Etiology 

Hydrocephalus is widely classified as communicating and non-communicating. Communicating hydrocephalus has no defect in CSF flow but rather is due to compromised CSF absorption with either over- or normal-production of CSF. Communicating type hydrocephalus is classically caused by post-hemorrhagic or post-inflammatory changes, commonly subarachnoid hemorrhage or bacterial meningitis complications [1]. Communicating hydrocephalus has been mainly understood as a deficit at the cranial arachnoid villi and granulation absorption site. However, the literature has provided evidence for the role of extracranial lymphatic vessels located in the ethmoid bone region. It has been shown that lymphatic absorption is decreased in a rat animal model of hydrocephalus. On the other hand, non-communicating hydrocephalus arises due to intraventricular CSF obstruction. The obstruction is commonly at the foramen of Monro, to the aqueduct of Sylvius, the fourth ventricle, or the foramen magnum. Non-communicating types can arise from hemorrhage, traumatic brain injury, brain tumor, cyst, or infection [7]. 

The CSF nourishes, protects, and removes toxic waste metabolites from the brain [1]. The leading production site of the CSF is the choroid plexus, with the cells connected via tight junctions. The blood-CSF barrier that forms protects the brain and serves as a shock absorber between the skull and the brain. Furthermore, CSF establishes homeostasis and composition consistency, facilitating proper neuronal functioning. This is an immune-privileged site and thus lacks substantial immunoglobulins. The central nervous system (CNS) immune cells, the microglial cells, are metabolically active and function to remove damaged or unused neuronal boutons [7]. 

Pathophysiology 

As noted before, hydrocephalus is defined as excessive CSF in the brain. The choroid plexus produces CSF within the lateral, third, and fourth ventricles. The fluid travels from the choroid plexus in the lateral ventricle, through the ventricular system, through the foramen of Monro to the third ventricle, and into the fourth ventricle via cerebral aqueduct or aqueduct of Sylvius. The CSF leaves the fourth ventricle laterally through the foramen of Luschka or medially through the foramen of Magendie into the subarachnoid space. CSF that travels through the foramen of Luschka will go to the cisternal or cerebral cortex subarachnoid space. Disruption in the flow of CSF, either by a mass or via absorption defect, plays a key role in the pathogenesis of hydrocephalus. 

Role of Extracranial Lymphatic Vessels 

**Figure 1:**
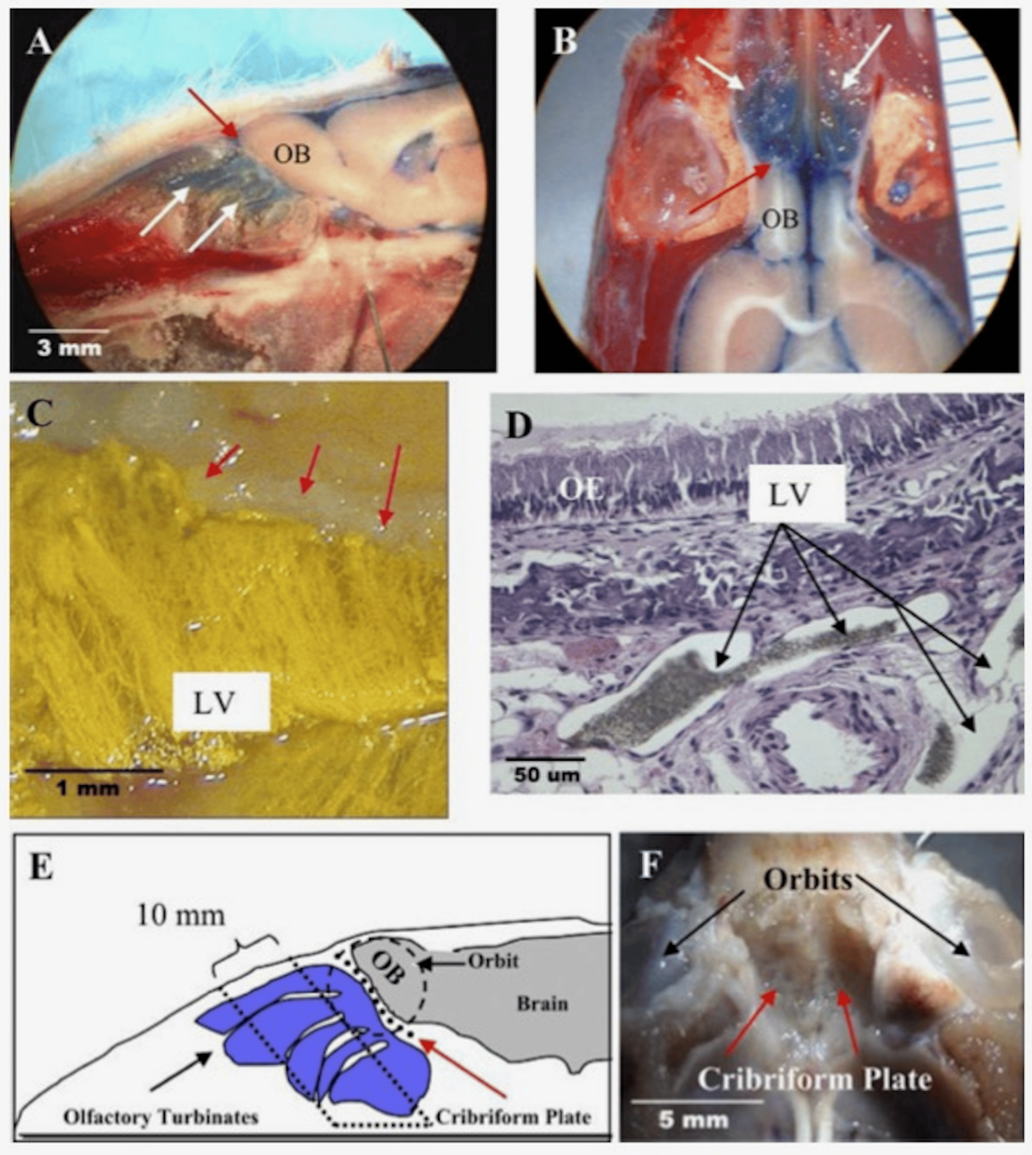
Transport of cerebrospinal fluid through perineural olfactory space into the olfactory turbinates in the rat Red arrow indicates the cribriform plate in A-C, E-F A and B) Evans blue dye in the turbinates (white arrows); C) Yellow Microfil depicting the lymphatic vessel highlighting the flow of CSF from the cistern magna subarachnoid space; D) Immunohistochemistry highlighting the lymphatic vessels of a neonatal rat; E) Schematic showing the sampling location to assess the role of CSF into the lymphatic vessel; F) Image of cribriform plate post-brain and olfactory bulb removal. CSF - cerebrospinal fluid Source: Nagra, 2010 [9]

CSF absorption primarily occurs at arachnoid granulations that protrude into dural venous sinuses, largely the superior sagittal sinus [1]. However, a previous study by Nagra et al. [8] has provided evidence that extracranial lymphatic vessels play a significant role in CSF absorption. Figure 1 allows for the visualization of CSF as it travels across the cribriform plate. Evans blue dye was injected into the CSF compartment, and it proceeded to distribute into the subarachnoid space around the olfactory bulbs to the cribriform plate (Figure 1-A, B). Additionally, the dye was also found external to the cranium and visualized within the olfactory turbinates (Figure 1-A, B). This shows that fluid within the CSF space travels rapidly throughout the cribriform plate. Moreover, the injection of Microfil into the CSF space was able to be visualized in olfactory submucosa within individual lymphatic vessels in the vast lymphatic network (Figure 1-C, D).

Furthermore, impairment of CSF absorption at the level of extracranial lymphatic vessels results in hydrocephalus [7,10]. 

Absorption pathways of CSF play a critical role in maintaining normal intracranial pressure. The primary pathway for CSF absorption is through the arachnoid villi and then is directed into the superior sagittal sinus. These arachnoid villi can be thought of as finger-like projections extending into the venous sinus, allowing for CSF reabsorption into the bloodstream [9]. 

Emerging evidence through animal studies suggests that extracranial lymphatic vessels at ethmoid turbinates play a role in CSF absorption. Qualitatively, the use of India Ink in rat models demonstrates structural channels connecting the cribriform plate to the nasal submucosa [11]. Similarly, studies utilizing the murine model injected Evan's blue high-contrast tracers into the CSF fluid and demonstrated CSF's ability to permeate the nasal lymphatic system [12]. Sheep and rabbit studies using mathematical modeling and various tracers injected into the CSF, such as 131I human serum albumin (HSA), India Ink, and indigo carmine, suggest that extracranial lymphatic vessels play a significant role in CSF drainage [11,13,14]. 

Through the use of yellow MicroFil injections, researchers demonstrated the connection between the subarachnoid space and nasal mucosa and turbinates in rat, pig, monkey, mice, and human cadaver studies [15]. Mathematical modeling has shown that 40-50% and greater than 50% of CSF drainage occurs through the olfactory pathway in rats, rabbits, and sheep, respectively [13,14,16]. While animal studies have shown extracranial lymphatic vessels at the ethmoid turbinates play a significant role in CSF absorption; further in-vivo studies in living humans are necessary. Human cadaver studies cannot accurately model the CSF fluid mechanics that occur in living beings. 

With respect to this concept, there has been evidence that the outflow resistance of CSF is impaired globally in kaolin rat models of communicating hydrocephalus. The decline in outflow resistance had a significant positive correlation to the severity of hydrocephalus, measured by an increased ventricular volume [9]. 

Role of Aquaporins

Various studies have pointed toward aquaporin channels playing a role in hydrocephalus. Aquaporins (AQPs) are water channels that facilitate water movement across membranes. AQP1 and AQP4 play a role in CSF production and water removal from the brain and, thus, have been implicated in the development of hydrocephalus [17].

Mice experiments have shown that AQP1 is associated with CSF production and management of intracranial pressure [7]. Measurements done evaluating water permeability of the choroid plexus between wild-type and AQP1 null mice showed that AQP1 deletion leads to a five-fold decrease in the osmotic gradient of the ventricular membrane [18]. AQP1, which is located on the choroid plexus epithelium and plays a role in CSF production, has been implicated in the development of hydrocephalus using the kaolin-induced hydrocephalus model in mice [19]. Mice with kaolin-induced hydrocephalus demonstrated a 50% reduction of AQP1 by endocytic retrieval and reduced ventricular size of the AQP-1 null mice [19]. 

Furthermore, AQP4 is located at the astrocyte foot processes of the blood-brain barrier and glial cells of the brain parenchyma [20]. A rat study involving Sprague-Dawley rats demonstrated increased AQP4 expression after mild hydrocephalus induction with kaolin injection [21]. There was also increased AQP4 expression in congenital hydrocephalus Texas rats [21]. However, Wistar-Hannover rats demonstrated no change in AQP4 expression [22]. In humans, cortical brain biopsies in patients with chronic hydrocephalus showed increased AQP4 immunoreactivity compared to controls [7,23]. The increase in AQP1 and AQP4 expression during hydrocephalus can be a compensatory mechanism to increase fluid clearance in the brain [20]. 

Regarding the role of AQP4 in the CNS, with respect to the glymphatic system, recent advancements have been made, specifically looking at its involvement in the pathogenesis of multiple CNS pathologies. The role of AQP4 in relation to the glymphatic system is multifold. It regulates CSF flow and clears metabolic cellular byproducts, thus supporting brain homeostasis. The aim of this paper is not to explore the glymphatic aquaporin role in the pathogenesis of hydrocephalus; however, this is an interesting clinical therapeutic target. 

Surgical interventions

This section will discuss current surgical interventions, of which ventricular shunt placement is the gold-standard therapy. 

Ventricular Shunting

Traditionally, the gold standard treatment option for hydrocephalus involves the placement of a ventricular shunt that will redirect CSF flow to another part of the body for absorption [7]. The shunt is a ventricular catheter attached to a valve attached to a distal catheter [24]. The three most common shunts are ventriculoperitoneal, ventriculopleural, and ventriculoatrial, with the ventriculoperitoneal shunt most frequently used [25]. The ventriculoperitoneal shunt usually guides CSF from the lateral ventricle into the peritoneum. This shunt is advantageous in children as the distal peritoneal end can be left long and does not require adjustment as the child grows [1]. The ventriculoatrial shunt pushes CSF through the jugular vein and is then directed into the superior vena cava, followed by the right atrium [1]. It is preferred for patients with peritonitis, extensive abdominal surgery, morbid obesity, or a history of ventriculoperitoneal shunt infection [1,7]. Due to anatomical challenges requiring specialized surgical expertise, a ventriculopleural shunt is typically only used if the other shunt types fail [1]. 

Endoscopic Third Ventriculostomy (ETV)

However, shunting is not the only intervention in this class for hydrocephalus. An alternative to shunting is endoscopic third ventriculostomy (ETV). ETV involves opening the floor of the third ventricle to allow the accumulated CSF to enter the prepontine basal cistern [1,7]. This is typically the procedure of choice for patients with aqueductal stenosis to avoid permanent shunt [1]. Certain risk factors have been identified that predispose to ETV failure, including young age, non-obstructive etiology, or shunt presence [4]. However, Zaben et al. [26] illuminate that even though there is a lower ETV success rate in lower age groups (44.4% vs. 66.7%), there is a comparable safety profile independent of age, thus indicating that ETV remains a viable option for infants one year or younger with obstructive hydrocephalus. 

Additional research has been conducted exploring the viability of ETV with choroid plexus cauterization (CPC). CPC is an innovative approach introduced in 1910 and reinstated as a viable technique by Dr. Benjamin Warf [27]. In infants under one year old, Warf demonstrated the utility of ETV with CPC in treating hydrocephalus [27]. He illuminated that EVT/CPC was more successful than solely ETV in this age population and is a top choice for infants with non-post-infectious hydrocephalus or myelomeningocele. However, a meta-analysis found no overall benefit to ETV/CPC versus ETV alone, but they did indicate that subgroup analysis demonstrated benefit in sub-Saharan African populations [28]. 

Furthermore, in 2020, the Hydrocephalus Clinical Research Network expanded on the research, revealing higher success rates for CSF shunt insertion (72% at six months and 65% at 12 months) than ETV/CPC (52% at 6 and 12 months) for infants in first-round hydrocephalus treatment [29]. Three to five years after the initial treatment, the ETV group showed fewer surgical revisions than those with CSF shunt insertion, but this was a non-statistically significant finding. Patients with ETV/CPC for the first treatment had higher mean hydrocephalus-related revision surgeries than those with ETV alone or CSF shunt insertion over the first year post-surgery. This group suggested a time-dependent benefit of ETV over CSF shunting [29]. 

Generally, these studies illuminate the need for further trials and studies to investigate the efficacy of CPC and ETV therapy in various patient populations and medically underserved areas and weigh the advantages and disadvantages. 

Problems With Traditional Surgical Interventions

It is important to note that permanent CSF interventions are often associated with a high failure risk and can involve re-intervention [4]. The risk of surgical infection is approximately 10%, and shunt failure rates are around 21%-42% in the first year post-placement [30]. In the first two years, shunt failure in children can be at a frequency of up to 50% [4]. Furthermore, each shunt failure necessitates at least one additional operation, increasing morbidity and mortality risks [4]. Additionally, there is a risk of shunt obstruction via flow blockage or infection due to biofilm formation, commonly seen with *Staphylococcus epidermidis *and *Staphylococcus aureus* [25,31]. 

A recent case report [30] shared a successful first-in-human treatment with a miniature biomimetic transdural shunt. This novel minimally invasive endovascular shunt mimics the function of arachnoid granulation to filter CSF from the CNS into the intracranial venous sinus network. This valved shunt has transdural deployment at the inferior petrosal sinus. The typical reabsorption of CSF flow was restored through the cerebellopontine angle cistern to the inferior petrosal sinus. The team utilized this novel approach on one octogenarian (age range 80-89) patient with subarachnoid hemorrhage from a ruptured middle cerebral aneurysm. An external ventricular drain (EVD) was placed and showed decreased frontal and lateral horns and third ventricle size, as demonstrated through a post-procedure MRI. The imaging also showed normal venous blood flow through the inferior petrosal sinus and internal jugular vein around the implant. This successful catheter-based treatment is a major development in establishing a more simplistic, non-invasive surgical intervention for patients requiring shunting treatment. However, this was a first-in-human study, and thus, further testing is necessary to establish long-term efficacy, safety, and applicable patient population. 

Non-surgical treatment

The following will talk about non-surgical interventions, most of which are still at the research level. 

Acetazolamide 

One frequently discussed treatment option for hydrocephalus is the carbonic anhydrase inhibitor acetazolamide [32]. The choroid plexus has high levels of carbonic anhydrase, so acetazolamide will directly inhibit CSF production [32]. However, studies have shown that the benefit of acetazolamide in hydrocephalic children is negligible [32].

Furthermore, there is high clinical certainty that the combination of acetazolamide and furosemide is not seen as a viable treatment option for post-hemorrhagic hydrocephalus in infants [32,33]. Two studies reported that preterm infants with post-hemorrhagic hydrocephalus treated with dual therapy had a higher risk of neurological complications, morbidity, and mortality [33]. Additionally, the International Post-Hemorrhagic Ventricular Dilatation (PHVD) Drug Trial Group established increased rates of shunt placement in those treated with acetazolamide and furosemide and increased morbidity (84% vs. 60%) compared to the standard approach [33,34].

However, in adults, acetazolamide was used instead of shunting, and it decreased intracranial pressure in cases of normal pressure hydrocephalus (NPH), a type of communicating hydrocephalus [7]. Moreover, it has been established as a good predictor of shunting response in NPH patients [35].

Memantine

More recently, memantine has become a drug of discussion surrounding hydrocephalus treatment. Memantine is a selective, non-competitive antagonist of the N-methyl-D-aspartate (NMDA) receptor, which has a neuroprotective contribution to neurological disease [36,37]. A previous study [36] explored the role of memantine therapy on neurologic and behavioral outcomes in kaolin-induced hydrocephalus rats. Memantine therapy stabilized ventricular enlargement with some behavioral improvement but did not reduce brain tissue changes. 

Another team [37] explored the neuroprotective effect of memantine in hydrocephalus-induced Wistar rats treated with or without a ventricular-subcutaneous shunt. Memantine treatment had significant improvement in sensorimotor development, spatial memory preservation, and astrocytic reaction reduction in the corpus callosum, cortex, and germinal matrix. When memantine was treated with shunt placement, there was a reduction in the cell death cascade. There is a promising response with memantine treatment that has the potential to assist in hydrocephalus treatment with or without other procedures, but more research must be completed. 

TRPV4 Antagonist

Transient receptor potential vanilloid 4 (TRPV4) is a nonselective, Ca2+-permeable channel expressed in the choroid plexus and facilitates ion flow across the epithelial membrane, influencing water movement and CSF production. It is known as a hub protein as it is activated by and integrates many stimuli. TRPV4 can be activated chemically via inflammatory mediators (arachidonic acid metabolites or cytokines) or physically via mechanical (pressure) and osmotic changes - mechanisms also known to cause hydrocephalus. 

A study utilizing a genetic rat model of communicating hydrocephalus showed that two different TRPV4 antagonists inhibited ventriculomegaly development [38]. Their data supported the relationship between TRPV4 and hydrocephalus development from cell surface expression or activation changes. Furthermore, the TRPV4 antagonists did not change ventricular volumes in normal animals and thus were mainly applicable in the hydrocephalic rats. Through the role of TRPV4 in regulating transepithelial ion flux and cell permeability in the choroid plexus, there is the possible utility of TRPV4 antagonists for most forms of hydrocephalus. Still, the idea of TRPV4 antagonist as a treatment measure is a new idea that requires much further examination due to multiple compounding factors, including electrolyte balance and fluid flux complexities. 

Furthermore, Toft-Bertelsen et al. [39] demonstrated lysophosphatidic acid (LPA) elevation in patients with subarachnoid hemorrhage and intraventricular hemorrhage-induced rat models. Elevated LPA directly impacted TRPV4, which activates the WNK/SPAK signaling path that promotes NKCC1-mediated CSF hypersecretion and ventricular enlargement. SPAK is a highly expressed kinase in the choroid plexus and a vital regulator of NKCC1. It was concluded that LPA is a lipid agonist of TRPV4 that facilitates the SPAK and NKCC1 pathways, leading to increased CSF secretion. This fundamental knowledge suggests the need for additional studies on potential therapies targeting LPA, TRPV4, SPAK, or NKCC1 to reduce hydrocephalus. 

NKCC1 and SPAK Inhibitors

The above discussion of TRPV4 and NKCC1 indicates their roles in maintaining CSF volume homeostasis and composition during disease states. While TRPV4 is activated due to osmotic changes, pressure, or inflammatory mediators, NKCC1 is activated by changes in electrolyte concentration. Furthermore, while different kinases activate TRPV4, NKCC1 is triggered primarily by WNK-SPAK and maintains cell volume levels and CSF composition [3]. 

One study [40] showed that NKCC1 phosphorylation in choroid plexus epithelium facilitates CSF hypersecretion by the NLRP3 inflammasome process. As part of the innate immune system, the NLRP3 inflammasome has been connected to neuroinflammation, and this study illuminated its function in hydrocephalus pathogenesis. 

Furthermore, Zhang et al. [41] designed and synthesized a non-ATP-competitive SPAK inhibitor called ZT-1a. This drug is unique in its development as the team used a "scaffold-hybrid" strategy, combining the pharmacophores from previously developed SPAK inhibitors (Closantel, Rafoxanide, and STOCK1S-14279). ZT-1a disrupted SPAK interaction with WNK, inhibited NKCC1 activity, and decreased CSF hypersecretion in post-hemorrhagic hydrocephalus models. With further study, this exciting innovation has strong therapeutic potential for hydrocephalus. 

Potential for Extracranial Lymphatic Vessels 

As noted, extracranial lymphatics play a crucial role in absorbing CSF. In the rat model of hydrocephalus, extracranial lymphatic vessel absorption was decreased. With respect​​ to this, a study that used a sheep hindlimb model observed the emergence of lymphedema following the removal of popliteal lymph nodes [42]. Similarly, Baker et al. induced lymphedema by excising a single popliteal lymph node along with its associated vessels [43]. About this, after inducing the lymph edema, this Johnston's group showed that an autologous lymph node transplantation lowered the lymph edema significantly. This is indirect evidence that enhancing lymphatic absorption can be used as a therapeutic strategy to deal with different pathologies.

Studies by Brunner et al. [44] demonstrate the importance of signaling molecules in lymphangiogenesis [45]. Specifically, their studies showed that VEGF-C to VEGFR3 modulates lymphatic vessel growth, particularly in the restructuring of the diabetic wound matrix. Interestingly, the use of viral vectors to increase vascular endothelial growth factor (VEGF)-C expression can improve both lymphatic and blood vessel growth. 

Furthermore, Choi et al. [46] showed that 9-cis retinoic acid can be applied therapeutically to facilitate lymphangiogenesis and lymphatic restoration. 

Applying this concept to the brain, substances such as VEGF-C/VEGFR3 and 9-cis retinoic acid can enhance the lymphatic uptake of the fluid and, if used locally, can induce lymphangiogenesis at the ethmoid bone level to help alleviate hydrocephalus. 

Discussion

Hydrocephalus, marked by ventricular expansion due to CSF accumulation, has a high burden of disease and cost of treatment that further accumulates with the high complication risks associated with the current standard of treatment. Management of this condition involves deviation of CSF with lumboperitoneal or ventriculoperitoneal shunts or the ETV technique. Placing a shunt in the brain through mini-laparotomy surgery can result in neurological complications, incisional abdominal herniation, and CSF pseudocyst formation [9]. Shunts provide symptomatic improvement, but one must consider the significant number of debilitating neurological deficits, high revision rates, mechanical failures, and infections, making this a poor treatment standard [9]. 

Nagra's thesis challenges the traditional view of hydrocephalus as a plumbing issue by drawing critical conclusions concerning the etiology of hydrocephalus, which can revolutionize hydrocephalus treatment [9]. With respect to this, the very conflict of accepting the textbook definition of CSF absorption via arachnoid villi and granulation was challenged. This was done by providing quantitative experimental data supporting extracranial lymphatic vessels' role at discrete anatomical locations. The CSF absorption impairment of these vessels contributes to hydrocephalus. Specifically, using the kaolin rat model of hydrocephalus, CSF outflow resistance was increased, and absorption was shown to be significantly decreased. Furthermore, to understand the molecular mechanical property of ventricular brain expansion, the Nagra et al. [47] study illustrated that β1-integrin and extracellular matrix interactions actively regulate interstitial fluid pressure. Any disruption to those interactions decreases brain parenchymal fluid pressure with respect to ventricles, creating a pressure gradient that leads to ventricular expansion. 

Nagra et al. [47] employed a micropipette servo-null pressure measuring system to investigate the active role of brain parenchyma in inducing ventriculomegaly. Two sets of experiments were conducted: one focused on measuring pressure in the lateral ventricle and adjacent parenchymal tissue before and after continuous injection of beta-integrin-blocking antibodies using a servo-nulling pressure measuring system. The second set involved injecting beta-integrin-blocking antibodies into the brain and assessing ventriculomegaly by brain sectioning after two weeks. The results showed a decline in periventricular pressures relative to pre-injection values when antibodies to beta-1 integrins were injected, whereas ventricular pressures were elevated and significantly higher than periventricular interstitial pressures. The estimated ventricular to periventricular pressure gradients reached up to 4.3 cm H2O. In chronic preparations, enlarged ventricles were observed in 72% of animals receiving anti-integrin antibodies but not in those receiving isotype control antibodies. This led to the conclusion that disrupting beta-1 integrin-matrix interactions generates pressure gradients favoring ventricular expansion, providing a plausible mechanism for hydrocephalus development [47]. 

This concept was formulated after considering that injecting anti-beta-1 integrin antibodies into the skin simulates inflammatory effects, leading to a significant drop in interstitial fluid pressure [48]. Although various causes and inflammatory mediators consistently induce this effect, what was particularly relevant to the Nagra et al. [47] study is that the injection of antibodies targeting alpha-2-beta-1 or beta-1 integrins can lower interstitial fluid pressure, indicating their key role in regulating this phenomenon [49]. This suggests that the interstitial matrix is dynamic, and disrupting its integrity can modify tissue pressure. Applying this concept to the brain, my group proposed that disturbances in beta-1 integrin function could be one of the defining factors leading to ventriculomegaly and offered a new perspective on hydrocephalus development.

Articulating the former, Nagra proposed the hypothesis that the extracellular matrix actively regulates interstitial fluid pressure rather than merely participating passively. Their data suggests that matrix components within the brain parenchyma exhibit similar dynamics to those observed in peripheral tissue function. Beta-1 integrins are believed to play crucial roles in central nervous system function and are expressed in choroidal and ependymal cells, glial cells, and vascular structures throughout the neuropil [50-52]. Astrocyte fibers, which surround particular microvessels in the adult primate, also express beta-1 integrins, as do the endothelial cells themselves [52]. Reduction of beta-1 integrin expression, specifically in neuronal and glial precursors, has been associated with convoluted cortex and reduced brain size [53]. Additionally, humans and mice with mutations in the laminin alpha-2 chain, an essential ligand for beta-1 integrins, exhibit disorganized cerebral cortices and ventricular dilation [54-56]. These findings suggest that disruption of beta-1 integrin-laminin interactions may contribute to pressure patterns and the development of hydrocephalus. 

It is worth noting that dystroglycan, another receptor sharing laminin as a primary matrix ligand with several beta-1 integrin receptors, has been associated with ventriculomegaly in dystroglycan-null mice [57]. The authors speculated that this might result from dysfunction of arachnoid granulations or stenosis of the cerebral aqueduct. However, it is plausible that the loss of dystroglycan, specifically in epiblasts, could affect cell-matrix interactions, similar to the injection of beta-1 integrin antibodies.

Understanding this etiology is useful when considering therapeutics useful for hydrocephalus treatment that can improve outcomes and limit side effects. It has been shown experimentally that decreased tissue pressure in the skin can be reversed with the anti-inflammatory agent α-trinositol [58,59]. Platelet-derived growth factor (PDGF-BB) isoform also can play a role by counteracting edema development through stimulation of αVβ3-integrins activity and adjusting extracellular matrix tension [60,61]. If these themes prove relevant to brain physiology, these could be valuable avenues of pharmacological treatment for hydrocephalus. 

Studies using magnetic resonance elastography (MRE) and diffusion tensor imaging (DTI) have provided quantitative data on brain tissue mechanics and support a decrease in compliance forces in a hydrocephalic brain and is proportional to the hydrocephalus severity [62,63]. Nagra et al. [47] demonstrated the role of integrin-matrix interactions in the development of hydrocephalus. Further, the data from human MRE and DTI studies hold up the experimental matrix integrity disruption concept [62]. Going forward, there is utility in exploring the role of anti-inflammatory agents, such as a-trinositol, or PDGF-BB, in individuals with hydrocephalus or post-shunt placement showing symptom improvement improving the extracellular matrix damage. Building upon MRE and DTI data and experimental support for matrix disorganization, Nagra proposed investigating whether administering these therapeutic agents to children with hydrocephalus or following shunt insertion could alleviate symptoms and reverse matrix damage. This hypothesis could be tested using imaging modalities, and it is anticipated that MRE and DTI data would show improvement in children receiving these agents. If successful, this approach could lead to the development of pharmacological treatments for hydrocephalus, reducing dependence on shunts.

## Conclusions

This paper shares the currently accepted and utilized techniques for hydrocephalus. Also, it discusses a shift in the understanding of hydrocephalus from a purely plumbing problem to an active involvement of the brain matrix in creating pressure gradients. The disruption of beta-1 integrin-matrix interactions and the resulting pressure gradients are proposed as key factors contributing to ventriculomegaly. These findings can help reduce the need for expensive, invasive, and, in some instances, hard-to-access therapies. By opening up new avenues for potential therapeutic interventions and raising the possibility of treating hydrocephalus with pharmacological agents, there is a potential for the reduced need for shunts. With high incidence rates, with most significant numbers in low-income countries, and elevated mortality rate if left untreated, there is an increased need for more accessible, lower-cost treatment plans based on mechanisms of hydrocephalus. There is still much to be explored regarding hydrocephalus's risk factors, etiology, and pathophysiology. Further research and clinical trials are required to validate these hypotheses and determine their clinical applicability. As studies expand, the knowledge gained must be considered and inform future treatment with a goal of decreased invasiveness, complications, and cost. We aspire to leverage the synergistic potential of lymphatic vessels combined with shunt therapy to alleviate the pathology of hydrocephalus.

## References

[1] Koleva M, De Jesus O (2023). Hydrocephalus. StatPearls [Internet].

[2] Zhang J, Williams MA, Rigamonti D (2006). Genetics of human hydrocephalus. J Neurol.

[3] Blazer-Yost BL (2023). Consideration of kinase inhibitors for the treatment of hydrocephalus. Int J Mol Sci.

[4] Hochstetler A, Raskin J, Blazer-Yost BL (2022). Hydrocephalus: historical analysis and considerations for treatment. Eur J Med Res.

[5] Dewan MC, Rattani A, Mekary R (2019). Global hydrocephalus epidemiology and incidence: systematic review and meta-analysis. J Neurosurg.

[6] Enslin JM, Thango NS, Figaji A, Fieggen GA (2021). Hydrocephalus in low and middle-income countries - progress and challenges. Neurol India.

[7] Agyei AA, Miles JD, Nagra G (2021). Rethinking hydrocephalus via interventional pathophysiology. Hydrocephalus: From Diagnosis to Treatment.

[8] Nagra G, Koh L, Zakharov A, Armstrong D, Johnston M (2006). Quantification of cerebrospinal fluid transport across the cribriform plate into lymphatics in rats. Am J Physiol Regul Integr Comp Physiol.

[9] Nagra G, Wagshul ME, Rashid S, Li J, McAllister JP 2nd, Johnston M (2010). Elevated CSF outflow resistance associated with impaired lymphatic CSF absorption in a rat model of kaolin-induced communicating hydrocephalus. Cerebrospinal Fluid Res.

[10] (2010). Extracellular fluid systems in the brain and the pathogenesis of hydrocephalus. https://tspace.library.utoronto.ca/bitstream/1807/26309/5/Nagra_Gurjit_201011_PhD_thesis.pdf.

[11] Kida S, Pantazis A, Weller RO (1993). CSF drains directly from the subarachnoid space into nasal lymphatics in the rat. Anatomy, histology and immunological significance. Neuropathol Appl Neurobiol.

[12] Norwood JN, Zhang Q, Card D, Craine A, Ryan TM, Drew PJ (2019). Anatomical basis and physiological role of cerebrospinal fluid transport through the murine cribriform plate. Elife.

[13] Boulton M, Flessner M, Armstrong D, Hay J, Johnston M (1997). Lymphatic drainage of the CNS: effects of lymphatic diversion/ligation on CSF protein transport to plasma. Am J Physiol.

[14] Boulton M, Flessner M, Armstrong D, Mohamed R, Hay J, Johnston M (1999). Contribution of extracranial lymphatics and arachnoid villi to the clearance of a CSF tracer in the rat. Am J Physiol.

[15] Johnston M, Zakharov A, Papaiconomou C, Salmasi G, Armstrong D (2004). Evidence of connections between cerebrospinal fluid and nasal lymphatic vessels in humans, non-human primates and other mammalian species. Cerebrospinal Fluid Res.

[16] Silver I, Kim C, Mollanji R, Johnston M (2002). Cerebrospinal fluid outflow resistance in sheep: impact of blocking cerebrospinal fluid transport through the cribriform plate. Neuropathol Appl Neurobiol.

[17] Carbrey JM, Agre P (2009). Discovery of the aquaporins and development of the field. Aquaporins.

[18] Oshio K, Watanabe H, Song Y, Verkman AS, Manley GT (2005). Reduced cerebrospinal fluid production and intracranial pressure in mice lacking choroid plexus water channel Aquaporin-1. FASEB J.

[19] Wang D, Nykanen M, Yang N, Winlaw D, North K, Verkman AS, Owler BK (2011). Altered cellular localization of aquaporin-1 in experimental hydrocephalus in mice and reduced ventriculomegaly in aquaporin-1 deficiency. Mol Cell Neurosci.

[20] Verkman AS, Tradtrantip L, Smith AJ, Yao X (2017). Aquaporin water channels and hydrocephalus. Pediatr Neurosurg.

[21] Paul L, Madan M, Rammling M, Chigurupati S, Chan SL, Pattisapu JV (2011). Expression of aquaporin 1 and 4 in a congenital hydrocephalus rat model. Neurosurgery.

[22] Aghayev K, Bal E, Rahimli T, Mut M, Balci S, Vrionis F, Akalan N (2012). Aquaporin-4 expression is not elevated in mild hydrocephalus. Acta Neurochir (Wien).

[23] Skjolding AD, Rowland IJ, Søgaard LV, Praetorius J, Penkowa M, Juhler M (2010). Hydrocephalus induces dynamic spatiotemporal regulation of aquaporin-4 expression in the rat brain. Cerebrospinal Fluid Res.

[24] Fowler JB, De Jesus O, Mesfin FB (2023). Ventriculoperitoneal Shunt. https://www.ncbi.nlm.nih.gov/books/NBK459351/.

[25] Hanak BW, Bonow RH, Harris CA, Browd SR (2017). Cerebrospinal fluid shunting complications in children. Pediatr Neurosurg.

[26] Zaben M, Manivannan S, Sharouf F, Hammad A, Patel C, Bhatti I, Leach P (2020). The efficacy of endoscopic third ventriculostomy in children 1 year of age or younger: a systematic review and meta-analysis. Eur J Paediatr Neurol.

[27] Warf BC (2005). Comparison of endoscopic third ventriculostomy alone and combined with choroid plexus cauterization in infants younger than 1 year of age: a prospective study in 550 African children. J Neurosurg.

[28] Ellenbogen Y, Brar K, Yang K, Lee Y, Ajani O (2020). Comparison of endoscopic third ventriculostomy with or without choroid plexus cauterization in pediatric hydrocephalus: a systematic review and meta-analysis. J Neurosurg Pediatr.

[29] Pindrik J, Riva-Cambrin J, Kulkarni AV (2020). Surgical resource utilization after initial treatment of infant hydrocephalus: comparing ETV, early experience of ETV with choroid plexus cauterization, and shunt insertion in the Hydrocephalus Clinical Research Network. J Neurosurg Pediatr.

[30] Lylyk P, Lylyk I, Bleise C (2022). First-in-human endovascular treatment of hydrocephalus with a miniature biomimetic transdural shunt. J Neurointerv Surg.

[31] Gutierrez-Murgas Y, Snowden JN (2014). Ventricular shunt infections: immunopathogenesis and clinical management. J Neuroimmunol.

[32] Del Bigio MR, Di Curzio DL (2016). Nonsurgical therapy for hydrocephalus: a comprehensive and critical review. Fluids Barriers CNS.

[33] Mazzola CA, Choudhri AF, Auguste KI, Limbrick DD Jr, Rogido M, Mitchell L, Flannery AM (2014). Pediatric hydrocephalus: systematic literature review and evidence-based guidelines. Part 2: management of posthemorrhagic hydrocephalus in premature infants. J Neurosurg Pediatr.

[34] International PHVD Drug Trial Group (1998). International randomised controlled trial of acetazolamide and furosemide in posthaemorrhagic ventricular dilatation in infancy. The Lancet.

[35] Miyake H, Ohta T, Kajimoto Y, Deguchi J (1999). Diamox((R)) challenge test to decide indications for cerebrospinal fluid shunting in normal pressure hydrocephalus. Acta Neurochir (Wien).

[36] Di Curzio DL, Nagra G, Mao X, Del Bigio MR (2018). Memantine treatment of juvenile rats with kaolin-induced hydrocephalus. Brain Res.

[37] Beggiora PD, da Silva SC, Rodrigues KP (2022). Memantine associated with ventricular-subcutaneous shunt promotes behavioral improvement, reduces reactive astrogliosis and cell death in juvenile hydrocephalic rats. J Chem Neuroanat.

[38] Hochstetler AE, Smith HM, Preston DC (2020). TRPV4 antagonists ameliorate ventriculomegaly in a rat model of hydrocephalus. JCI Insight.

[39] Toft-Bertelsen TL, Barbuskaite D, Heerfordt EK (2022). Lysophosphatidic acid as a CSF lipid in posthemorrhagic hydrocephalus that drives CSF accumulation via TRPV4-induced hyperactivation of NKCC1. Fluids Barriers CNS.

[40] Zhang Z, Tan Q, Guo P (2022). NLRP3 inflammasome-mediated choroid plexus hypersecretion contributes to hydrocephalus after intraventricular hemorrhage via phosphorylated NKCC1 channels. J Neuroinflammation.

[41] Zhang J, Bhuiyan MI, Zhang T (2020). Modulation of brain cation-Cl(-) cotransport via the SPAK kinase inhibitor ZT-1a. Nat Commun.

[42] Tobbia D, Semple J, Baker A, Dumont D, Semple A, Johnston M (2009). Lymphedema development and lymphatic function following lymph node excision in sheep. J Vasc Res.

[43] Baker A, Kim H, Semple JL, Dumont D, Shoichet M, Tobbia D, Johnston M (2010). Experimental assessment of pro-lymphangiogenic growth factors in the treatment of post-surgical lymphedema following lymphadenectomy. Breast Cancer Res.

[44] Brunner LM, He Y, Cousin N (2023). Promotion of lymphangiogenesis by targeted delivery of VEGF-C improves diabetic wound healing. Cells.

[45] Shimizu Y, Che Y, Murohara T (2023). Therapeutic lymphangiogenesis is a promising strategy for secondary lymphedema. Int J Mol Sci.

[46] Choi I, Lee S, Kyoung Chung H (2012). 9-cis retinoic acid promotes lymphangiogenesis and enhances lymphatic vessel regeneration: therapeutic implications of 9-cis retinoic acid for secondary lymphedema. Circulation.

[47] Nagra G, Koh L, Aubert I, Kim M, Johnston M (2009). Intraventricular injection of antibodies to beta1-integrins generates pressure gradients in the brain favoring hydrocephalus development in rats. Am J Physiol Regul Integr Comp Physiol.

[48] Wiig H, Rubin K, Reed RK (2003). New and active role of the interstitium in control of interstitial fluid pressure: potential therapeutic consequences. Acta Anaesthesiol Scand.

[49] Reed RK, Rubin K, Wiig H, Rodt SA (1992). Blockade of beta 1-integrins in skin causes edema through lowering of interstitial fluid pressure. Circ Res.

[50] Grooms SY, Terracio L, Jones LS (1993). Anatomical localization of beta 1 integrin-like immunoreactivity in rat brain. Exp Neurol.

[51] Paulus W, Baur I, Schuppan D, Roggendorf W (1993). Characterization of integrin receptors in normal and neoplastic human brain. Am J Pathol.

[52] del Zoppo GJ, Milner R (2006). Integrin-matrix interactions in the cerebral microvasculature. Arterioscler Thromb Vasc Biol.

[53] Graus-Porta D, Blaess S, Senften M (2001). Β1-class integrins regulate the development of laminae and folia in the cerebral and cerebellar cortex. Neuron.

[54] Sunada Y, Edgar TS, Lotz BP, Rust RS, Campbell KP (1995). Merosin-negative congenital muscular dystrophy associated with extensive brain abnormalities. Neurology.

[55] Philpot J, Cowan F, Pennock J, Sewry C, Dubowitz V, Bydder G, Muntoni F (1999). Merosin-deficient congenital muscular dystrophy: the spectrum of brain involvement on magnetic resonance imaging. Neuromuscular Disorders.

[56] Miyagoe-Suzuki Y, Nakagawa M, Takeda S (2000). Merosin and congenital muscular dystrophy. Microsc Res Tech.

[57] Satz JS, Barresi R, Durbeej M, Willer T, Turner A, Moore SA, Campbell KP (2008). Brain and eye malformations resembling Walker-Warburg syndrome are recapitulated in mice by dystroglycan deletion in the epiblast. J Neurosci.

[58] Lund T, Reed RK (1994). Alpha-Trinositol inhibits edema generation and albumin extravasation in thermally injured skin. J Trauma Acute Care Surg.

[59] Rodt SA, Reed RK, Ljungström M, Gustafsson TO, Rubin K (1994). The anti-inflammatory agent alpha-trinositol exerts its edema-preventing effects through modulation of beta 1 integrin function. Circ Res.

[60] Rodt SA, Ahlén K, Berg A, Rubin K, Reed RK (1996). A novel physiological function for platelet-derived growth factor-BB in rat dermis. J Physiol.

[61] Lidén A, Berg A, Nedrebø T, Reed RK, Rubin K (2006). Platelet-derived growth factor BB-mediated normalization of dermal interstitial fluid pressure after mast cell degranulation depends on beta3 but not beta1 integrins. Circ Res.

[62] Tan K, Meiri A, Mowrey WB (2018). Diffusion tensor imaging and ventricle volume quantification in patients with chronic shunt-treated hydrocephalus: a matched case-control study. J Neurosurg.

[63] Aunan-Diop JS, Pedersen CB, Halle B (2022). Magnetic resonance elastography in normal pressure hydrocephalus-a scoping review. Neurosurg Rev.

